# An optimized model based on adaptive convolutional neural network and grey wolf algorithm for breast cancer diagnosis

**DOI:** 10.1371/journal.pone.0304868

**Published:** 2024-08-19

**Authors:** Khaled Alnowaiser, Abeer Saber, Esraa Hassan, Wael A. Awad

**Affiliations:** 1 College of Computer Engineering and Sciences, Prince Sattam Bin Abdulaziz University, Al Kharj, Saudi Arabia; 2 Information Technology Department, Faculty of Computers and Artificial Intelligence, Damietta University, Damietta, Egypt; 3 Faculty of Artificial Intelligence, Kafrelsheikh University, Kafrelsheikh, Egypt; 4 Computer Science Department, Faculty of Computers and Artificial Intelligence, Damietta University, Damietta, Egypt; University of Mauritius, MAURITIUS

## Abstract

Medical image classification (IC) is a method for categorizing images according to the appropriate pathological stage. It is a crucial stage in computer-aided diagnosis (CAD) systems, which were created to help radiologists with reading and analyzing medical images as well as with the early detection of tumors and other disorders. The use of convolutional neural network (CNN) models in the medical industry has recently increased, and they achieve great results at IC, particularly in terms of high performance and robustness. The proposed method uses pre-trained models such as Dense Convolutional Network (DenseNet)-121 and Visual Geometry Group (VGG)-16 as feature extractor networks, bidirectional long short-term memory (BiLSTM) layers for temporal feature extraction, and the Support Vector Machine (SVM) and Random Forest (RF) algorithms to perform classification. For improved performance, the selected pre-trained CNN hyperparameters have been optimized using a modified grey wolf optimization method. The experimental analysis for the presented model on the Mammographic Image Analysis Society (MIAS) dataset shows that the VGG16 model is powerful for BC classification with overall accuracy, sensitivity, specificity, precision, and area under the ROC curve (AUC) of 99.86%, 99.9%, 99.7%, 97.1%, and 1.0, respectively, on the MIAS dataset and 99.4%, 99.03%, 99.2%, 97.4%, and 1.0, respectively, on the INbreast dataset.

## 1. Introduction

### 1.1 Overview

Cancer, as a group of diseases, occurs when cells divide indefinitely and spread into surrounding tissue, accumulating to form a lump known as a tumor or malignancy. When the body needs new cells for repair or replacement, normal cells in the human body divide to produce those new cells. Over time, normal cells die, but cancerous cells exhibit abnormal behavior as a result of cell mutations that push out normal cells. Breast cancer (BC), which primarily affects adult females, is one of the more common cancer types [1].

BC is most likely the deadliest infection affecting women worldwide. The improper growth of breast cells can lead to tumors in women. These enormous tumor cells divide into cancerous and non-cancerous cells depending on the area, size, and location. The term "benign" refers to the noncancerous tumor’s original tumor area, whereas "malignant" refers to the cancerous tumor’s secondary tumor area.

Benign tumors have no effect on the lives of women because they are treatable and can be prevented with the appropriate therapies. A malignant tumor can only be treated by receiving the required medical attention, like surgery or radiation. The disease classification contains categories such as tumor class or not, sporadic or one-time occurrence, and harmless or dangerous.

Stage 0 in BC is the most severe (carcinoma in situ). It then progresses from stage I (1) to IV (4) [2]. BC stages are described in Table 1.

**Table 1:** BC stages.

stages	Stage Description
Stage 0	In nearby tissues, abnormal cells are present but do not proliferate
Stage 1: In situ	Only the breast’s lobules and ducts contain cancerous cells.
Stage 2: Localized	A few lymph nodes are affected, and the tumor is only 20 to 50 mm in size.
Stage 3: Regional	Greater than 50 mm tumour size, skin or chest wall growth, and increased lymph node involvement
Stage 4: Distant	The tumor can grow and spread to any area of the body.

Early detection of BC contributes to a higher survival rate for this disease. As a result, regular screening is regarded as one of the most important tools for aiding in the early detection of this type of cancer. A mammogram is regarded as one of the most effective screening modalities for early detection of BC [3], as it can reveal various abnormalities in the breast even before symptoms appear. Several research for BC detection and classification have been proposed in an effort to build more effective CAD and diagnosis systems for BC, due to considerable advances in machine learning (ML) and image processing techniques.

Medical image processing techniques for histopathology images are evolving swiftly, but an automated approach is still crucial to achieving efficient and extremely precise results. Enhancing health systems is one of the uses of ML. The dynamic nature of tasks like pre-processing and feature extraction in traditional ML techniques reduces the system performance. In order to address the issues with conventional ML techniques, the idea of DL has been applied to extract pertinent features from the images and use it for classification purpose [4, 5].

The CNNs are the most commonly utilized DL algorithms that have been suggested in the literature. With the 2D input-image structure, the CNN architecture has been specially modified [4, 6–11]. A CNN training task needs a lot of data, which the medical field, especially in BC, lacks. This issue can be resolved by applying a Transfer Learning (TL) technique.

### 1.2 Problem statement

About 15% of all female cancers in the world are carcinomas of the breast. In the United States, 1,735,350 new cases of cancer are anticipated, and 609,640 fatalities are anticipated in 2018. An estimated 878,980 women will develop cancer, of which 266,120 will likely develop BC and cause 40,920 deaths [12]. In 2020, there will have been 134 632 new cases of cancer in Egypt, 22 038 of which will have been BC, with 9 148 of those cases resulting in death [1]. Despite an increase in BC incidence, the mortality rate is trending downward because more people are using better diagnostic tools and getting better care for the disease [13]. However, it produced enormous amounts of diagnostic data from the screened women’s medical records, including mammograms, ultrasounds, and biopsies. The limited number of professionals who are available is also delaying the detection of cancer. CAD technologies can be useful in this situation and raise diagnostic accuracy. CAD systems for mammography are either utilized as a visual aid to help radiologists or as a second opinion [14].

Different methods have been explored to categorize BC. However, there is still a need to create and apply a suitable strategy for a BC diagnosis system that is more successful.

### 1.3 Paper contribution

The main motivating factors for this paper are summarized below.

- Improve the network classification results using the SVM classifier.
- Remove non-breast regions in preprocessing steps to reduce the training computation time.
- Implement a CNN-based TL-BiLSTM network for early detection of breast tumors (BTs).
- Optimized the network parameters using the GWO algorithm.
- The SVM and RF classifiers are applied to improve the classification performance.
- Other DL models are compared with the recently presented model.
- The presented model is evaluated with standard metrics like precision, sensitivity, specificity, and accuracy.

### 1.4 Paper organization

The paper structure is as follows: Section 2 describes the related work, and Section 3 describes the proposed model for identifying and classifying BC using TL methods. The experimental results are compared to the actual data in Section 4. In Section 5, the paper is concluded.

## 2. Related works

The integration of ML and DL technologies in breast cancer diagnosis has significantly improved accuracy, surpassing even skilled clinicians. Utilizing ML and DL technologies can improve diagnosis accuracy by 91.1 percent, compared to a skilled clinician’s diagnosis accuracy of 79.9% [6]. CNN may only use 1 input layer, 28 hidden layers, and 1 output layer for BC detection, according to Ting et al. [15]. Toaçar et al. [16] implemented the BreastNet for pertinent data extraction from their BC dataset. The model accuracy attained by BreastNet (98.80%) is superior to VGG-16, VGG-19, and AlexNet. Abbas [17] classified breast mammography images using multi-layers CNN model. MIAS dataset was applied to evaluate the presented model and achieve values of 92%, 84.20%, 91.50%, and 0.91 for sensitivity, specificity, accuracy, and AUC, respectively. Sha et al. [18] proposed a deep CNN for BT classification and evaluated the presented model using MIAS dataset. The grasshopper optimization method was applied to enhance the suggested CNN layers. The enhanced networks perform at 96%, 93%, and 92% in terms of sensitivity, specificity, and accuracy, respectively. The CNN suggested by Charan et al. [19] has 13 layers (6 convolutions, 4 average-pooling, and 3 fully connected). The softmax function is used to classify an input image with dimensions of 224 224 x 3. This model was achieved 65% for accuracy using the MIAS dataset. Wahab et al. classified mitoses using the TL approach in [20]. Their demonstrated method produced accuracy of 0.50 and recall of 0.80. Lotter et al. [21] fine-tuned the ResNet-50 network for classifying BTs into five classes. For sensitivity, specificity, and AUC, the proposed method achieved 96.2%, 90.9%, and 0.94, respectively. Jiang et al. [22] transferred the learned parameters from GoogleNet and AlexNet models to classify BT. The presented model used the film mammography number 3 (BCDR-F03) dataset to evaluate it and achieved 0.88 and 0.83 for accuracy using GoogleNet and AlexNet, respectively. Khan et al. [23] employed a benchmark database to fine-tune GoogleNet, VGG-Net, and ResNet models. The model’s accuracy was 97.525%. The RF dissimilarity was employed by Cao et al. [24] to enhance TL results on ResNet-125. The model was put to the test on the "ICIAR 2018" dataset and achieved 82.90% for accuracy. To train a target breast model, Deniz et al. [25] used the AlexNet and VGG-16 parameters discovered on the BreaKHis dataset. Overall, their model had a 91.37 percent accuracy rate. Using the same dataset and the DenseNet-161 CNN, Celik et al. [26] achieved an accuracy of 91.57%. Abeer et al. [27] transferred the BT parameters from Inception-V3, VGG-16, and VGG-19. On the MIAS breast dataset, the suggested model is assessed. The results showed that the VGG-16 has a 96.8% overall accuracy in identifying and classifying BC. A new model was developed by Abeer et al. [28] using the TL method. The presented model is divided into two main sections. The five most widely pre-trained CNNs are used as the foundation for their model. The breast model is trained using the features that were extracted, with the exception of the final three layers. The MIAS is used to validate the proposed model and train the final layers. The overall scores for accuracy, sensitivity, specificity, and precision, as well as the F-score and AUC, are 98.96%, 97.83%, 99.13%, 97.35%, and 97.66%, respectively, using the VGG-16 network. Abeer et al. [29] demonstrated a DL methodology based on the TL for categorizing and detecting BT. They trained the VGG-16 and VGG-19 networks using the INbreast dataset. For accuracy, sensitivity, specificity, and AUC, the presented model received scores of 97.1%, 96.3%, 97.9%, and 0.988, respectively. On the same dataset, Abeer et al. [30] achieved 99.236%, 99.1%, 96%, 98.8%, and 0.998 for accuracy, specificity, precision, sensitivity, and AUC, respectively, using SqueezeNet, LSTM, and Adam optimizer. Based on a region-based CNN, Akselrod-Ballin et al. [31] assessed a DL model for BT classification using the INbreast dataset. The accuracy rate for the model that was provided was 78%. Khan et al. [23] developed a TL model for classifying BT using GoogleNet, VGG-Net, and ResNet, pre-trained models. This model achieved an accuracy score of 97.525 percent on a widely used benchmark dataset. Al-Antari et al. [32] used feedforward CNN, ResNet 50, and Inception ResNet-V2 CNNs to build a DL model to categorize BC. The proposed model’s accuracy reached 95.32% across the INbreast dataset. Raaj et al. [33] suggested hybrid CNN architecture in the MIAS dataset. The hybrid CNN architecture, radon transform, and data augmentation module make up the suggested system more accurate and achieve 98% sensitivity, 98.66% specificity, 99.17% accuracy. A new CNN was developed by El Houby et al. [34] for classifying BTs. The MIAS, INbreast, and DDSM datasets are utilized for the evaluation. Using the CLAHE algorithm, the picture contract is improved. For the MIAS database, the total sensitivity, specificity, accuracy, and AUC are 98%, 92.6%, 95.3%, and 0.974, respectively. In order to classify BC, Singh et al. [35] created and applied an ML framework. The INbreast dataset was used to evaluate the illustrated framework, which received scores of 88.0%, 90.4%, and 92.0% for specificity, accuracy, and sensitivity, respectively. A TL framework was implemented by Chakravarthy et al. [36] The final 3 layers from AlexNet, GoogleNet, ResNet-50, and Dense-Net 121 were left out of the given model, and these layers were trained using the INbreast dataset. The BT classes are categorized using the SVM classifier. The accuracy of this model that was presented was 96.6%.

The related literature has the Strengths of their proposed models: i) Improved Diagnosis Accuracy: ML and DL technologies such as BreastNet and ResNet-50 have significantly enhanced breast cancer diagnosis accuracy, reaching an impressive rate of 98% compared to skilled clinicians. ii) Robustness Across Datasets: The proposed models have been proven to be robust and effective in BC classification through extensive testing on various datasets like MIAS, INbreast, and DDSM. iii) Data Augmentation: Data augmentation techniques like the radon transform and CLAHE have been used to improve image quality and classification performance. However, there are some weaknesses in the proposed models:i) Model Complexity: Certain models, such as those with 28 hidden layers, may result in increased computational demands and training time. ii) Limited Explanation: DL models’ lack of interpretability hinders clinical adoption, as clinicians prefer transparent models due to their difficulty in understanding decision-making features. iii) Large Training Datasets: Large labelled datasets are crucial for effective training of deep learning models, which may not always be available, particularly for rare medical conditions. iv) Deployment in Clinical Settings: The integration of these models into clinical workflows and ensuring regulatory compliance can be a challenging and time-consuming task.

## 3. Proposed model

**Fig 1:**
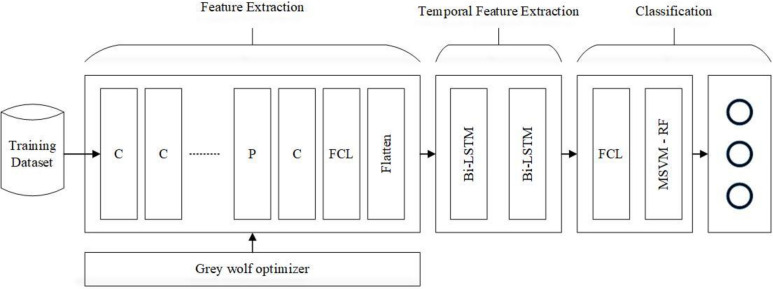
Shows the proposed model steps.

**Fig 2:**
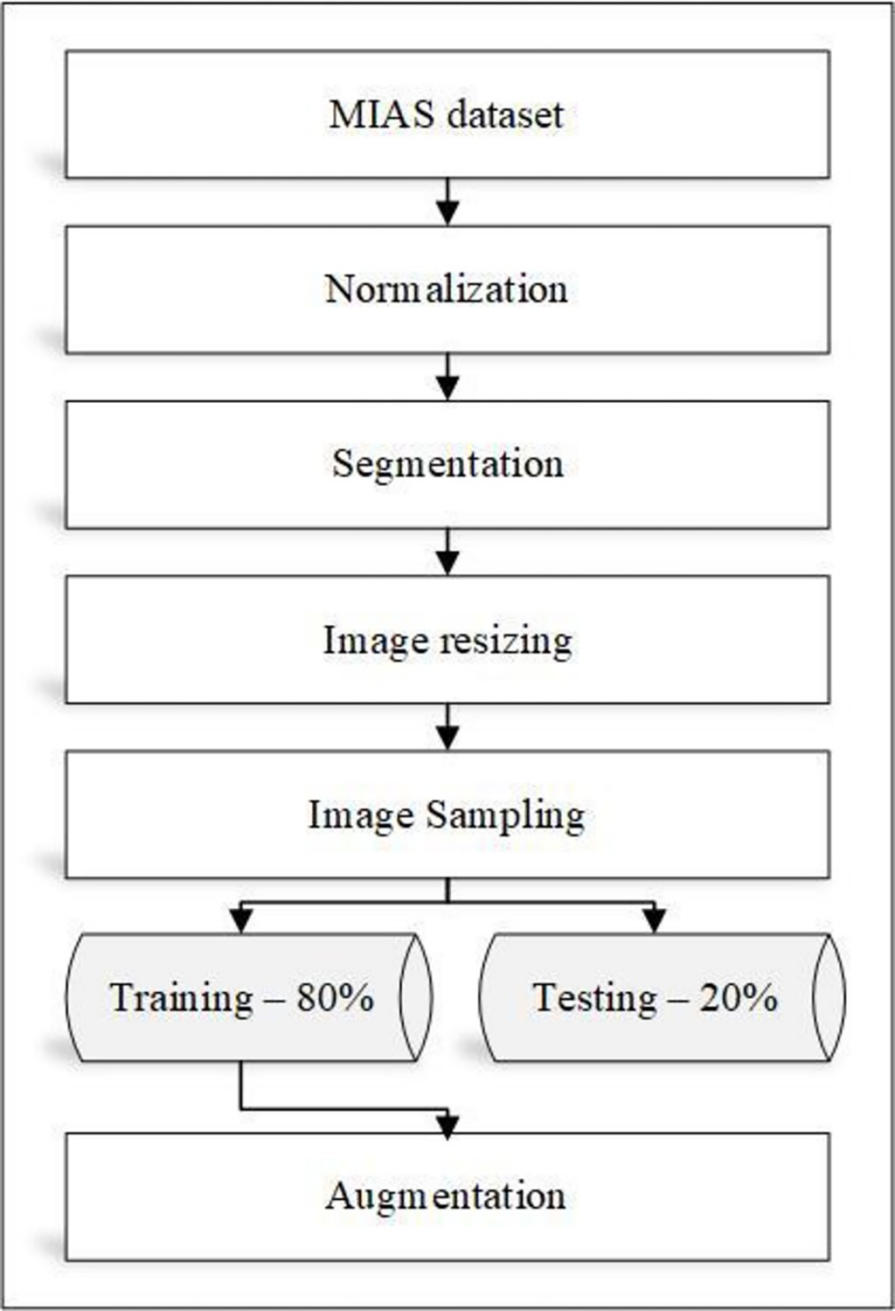
Shows the data pre-processing steps.

The model presented in this context has two primary components, as shown in Figs 1 and 2. First, the breast dataset is pre-processed, followed by the improved deep VGG-16 and DenseNet-121 pre-trained CNNs for extracting and classifying the breast features.

### 3.1 Data pre-processing

As illustrated in Fig 2, the Data Pre-processing phase contains six processes that improve image contrast, minimize computing time, and improve classification performance.

1. Normalization
  The process of normalization modifies the range of pixel intensity levels. Data normalization is a crucial stage since it guarantees that each input parameter has a comparable data distribution. While the network is being trained, this accelerates convergence.
2. Segmentation
  Through the use of a region-based segmentation technique, the tumor tissues have been identified. Region-based interventions focus on pixels with comparable properties. These techniques are easy to utilize and noise-proof. In an efficient seed pixel-based region-growing segmentation, surrounding pixels are evaluated and added to a region using similarity criteria. Up until no more pixels fit the criteria, the process is repeated.
  A segmentation-based method for automatic patch extraction may reduce computation time and concentrate the analysis on the area most impacted by cancer [37].
3. Image Resizing
  To correlate with the pre-trained models’ input size, the input data are shrunk to 224 × 224 and translated to 3 channels.
4. Image Sampling
  The MIAS dataset is split to 80%: 20% for training and testing, respectively.
5. Augmentation
  Large datasets are often required for effective training of CNNs. The majority of scenarios, however, as well as practical reasons, make it challenging to gather numerous medical datasets. In the context of CNN, artificial data augmentation is a strategy that is frequently used to expand the number of datasets while lowering overfitting. In order to assist the neural network generalize better, we can add some random data to the input. Less overfitting of a neural network during training can be caused by random noise.
  Adding random zero-mean gaussian white noise is used in this paper to enhance the amount of mammographic data by standard deviation (σ = 20, 30, 40, 50). The generated data is then horizontally flipped. As a result of this process, the data was multiplied by eight.

### 3.2 Transferring the learned features

The pre-trained CNNs networks VGG-16 and DenseNet- 121 are employed in this study and enhanced using grey wolf and Bi-LSTM for the BC classification process, as indicated in Fig 1. These CNNs are trained using the ImageNet dataset. Filters are utilized in the network layers to identify input properties like colors and horizontal and vertical lines. Small pieces and insignificant shapes can therefore be recognized. The resulting output can be used to identify the class (cats, birds, etc.) to which the input image is a part of.

**Fig 3:**
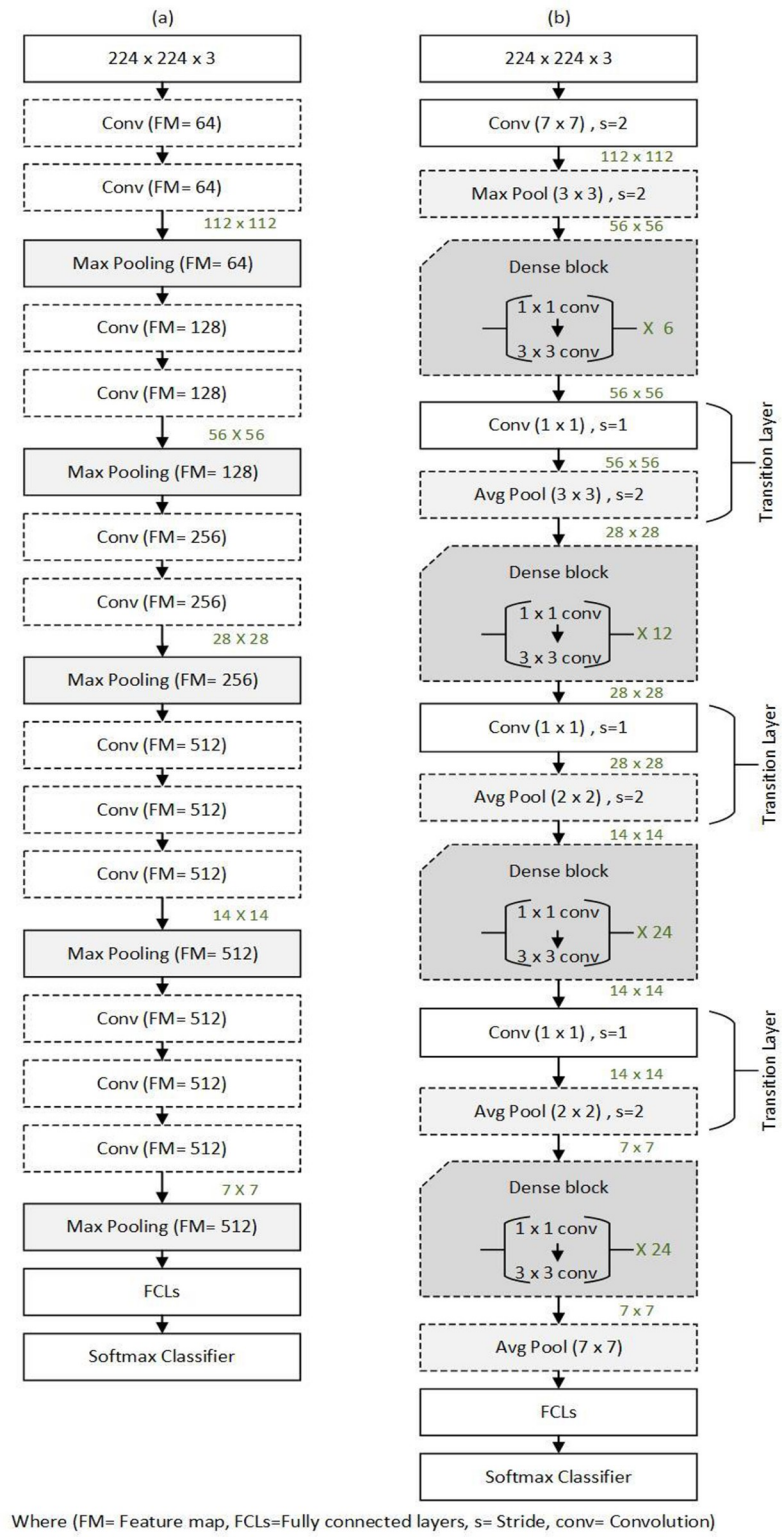
(a), depicts the construction of the VGG-16. (b), Illustrates the structure of the DenseNet-121.

The pre-trained network is subsequently used (in this paper for BT- classification) to categorize various items in a new dataset. The training parameters from the source task are communicated to the target task, as indicated in Fig 3, except for the final three layers (Fully Connected Layer (FCL), softmax, and categorization). The subsequent network training uses the retrieved patches from the pre-processing segmentation phase. Newly taught dense layers are therefore constrained. To further enhance the classification outcomes, layers from the pre-trained network are mixed with layers from the breast dataset.

The VGG-16 is trained using the ImageNet dataset. It has a complex yet straightforward architecture. It includes a softmax classifier, three FCLs, five max-pooling layers, and 13 convolution layers. 224 x 244 RGB makes up the input image.

The DenseNet architecture tries to enhance the depth of DL networks while simultaneously boosting training efficiency by employing shorter connections across layers. DenseNet-121 consists of 120 convolution layers and four Average Pooling layers.

In pre-trained networks, the learning layers’ characteristics are typically passed to the fully connected layer for classification. The proposed model creates a hybrid structure by adding the BiLSTM layer to the pre-trained networks. The proposed approach considerably improves the classifier’s performance in learning the temporal information because of the use of the BiLSTM network architecture. Two LSTMs are trained, one using the input sequence as it is and the other using a reverse copy of it. With BiLSTM, the relationship between previous outputs and existing inputs is addressed. This relationship is determined in two ways, from past to future and from future to past BiLSTM, which differs from LSTM in this regard, produces better outcomes and typically gathers information faster than LSTM. Since the BiLSTM algorithm is made to work with sequential data, preprocessed images are first used to build feature matrices (or feature vectors). The CNN network is the source of these matrices.

### 3.3 Grey wolf optimizer

The Grey Wolf Optimization (GWO) algorithm is a metaheuristic algorithm inspired by nature that mimics the natural leadership structure and hunting behavior of grey wolves. The algorithm simulates the leadership hierarchy using four different sorts of grey wolves: alpha, beta, delta, and omega. The GWO algorithm is created using a mathematical model of grey wolf hunting behavior.

The algorithm begins by initializing a population of grey wolves at random (possible solutions), and during the course of iterations, it calculates the likely location of the prey. The alpha, beta, and delta wolves lead the hunt, and the omega wolves trail these three wolves. The algorithm’s three primary hunting processes include tracking, pursuing, and approaching the prey, pursuing, encircling, and harassing the prey until it stops moving, and attacking the prey. The social hierarchy and hunting behavior of grey wolves are mathematically modeled to design the GWO algorithm [38].

## 4. Results

### 4.1 Mammographic dataset

**Fig 4:**
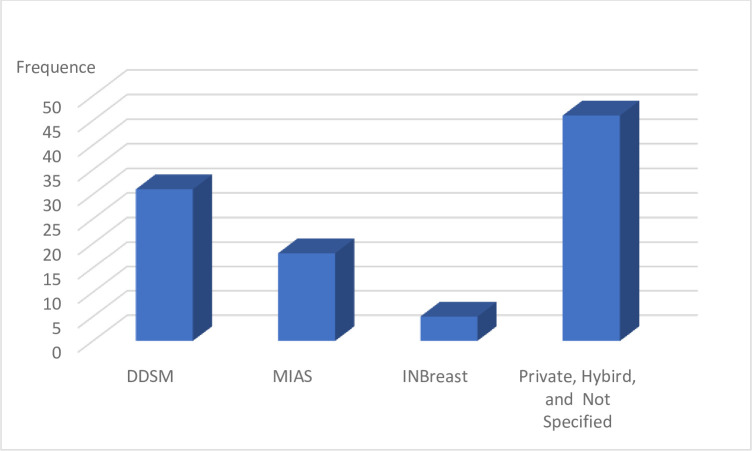
Shows the frequency of the most popular datasets that applied in BC classification.

**Fig 5:**
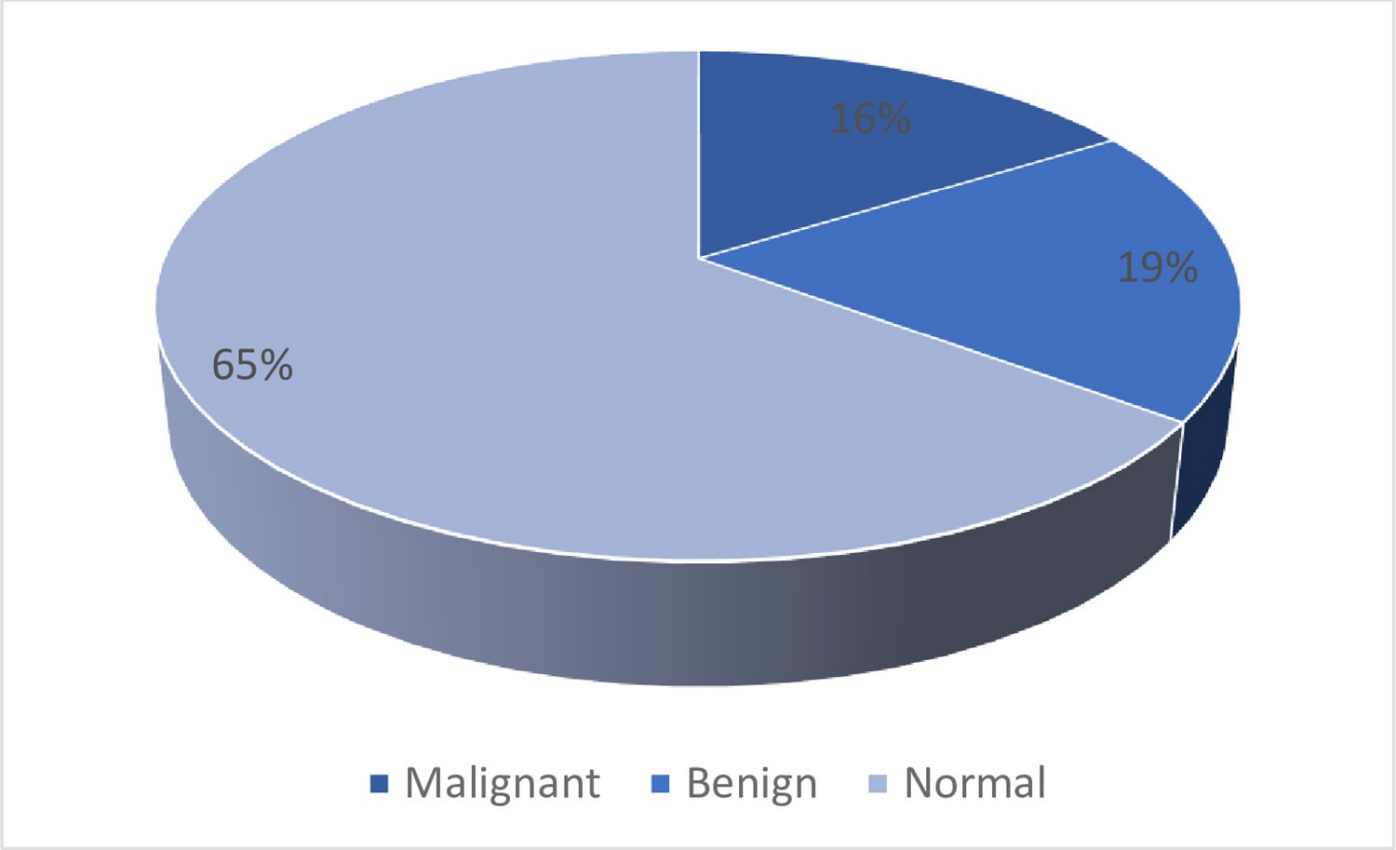
Describes the classes in the MIAS dataset.

**Fig 6:**
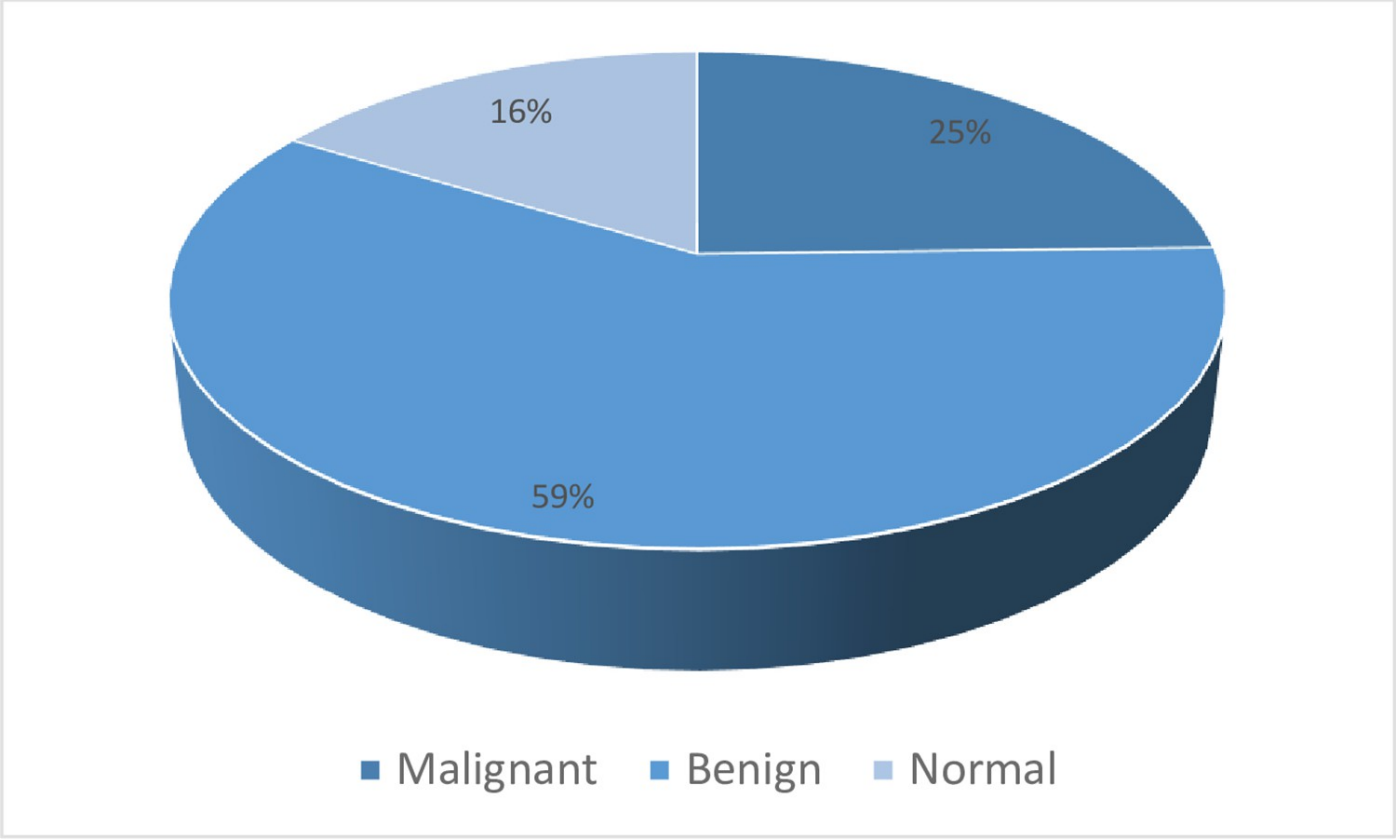
Describes the INbreast dataset classes.

The MIAS and INbreast datasets are applied in this paper to evaluate the proposed model. The applied datasets are the most familiar datasets applied in BT detection and classification systems as shown in Fig 4 [39]. MIAS contains 322 cases for 3 different classes (Malignant, Benign, and normal) in portable grey map (PGM) format and 1024 x 1024 in size. Fig 5 shows the different three classes in the MIAS dataset. While the INbreast dataset contains 410 images for the same classes determined by the Breast Imaging-Reporting and Data System value. The classes in the INbreast dataset are illustrated in Fig 6.

### 4.2 Experimental results

Precision, sensitivity, F measure, and accuracy are some of the measures that are frequently used to assess categorization performance. The true-positive (TP), false-positive (FP), true-negative (TN), and false-negative (FN) metrics are applied to measure each of them using Eqs 1–4. The result was incorrectly returned as affirmative despite TN indicating that it was negative. The test for TP was indeed positive when it was returned. In contrast, TP and TN denote that the result was returned as positive and is in fact positive.

$$Accuracy=\frac{TP}{TP+TN}$$ (1)

$$Sensitivity=\frac{TP}{TP+FN}$$ (2)

$$Specificity=\frac{TN}{TN+FP}$$ (3)

$$Precision=\frac{TP}{TP+FP}$$ (4)

Several experiments are discussed in this section for investigating the presented model performance on the MIAS. Here, features are extracted using two CNN models VGG-16 and DenseNet- 121 and finally classified using three different classification algorithms. The mentioned dataset was contained 3 different categories for Benign, Malignant, and Normal. The pre-processed dataset was split into 80%:20% for training and testing, respectively. The illustrated results in Tables 2–5 shows that the SVM classifier obtain better results than softmax and RF in almost all variables.

**Table 2:** The MIAS dataset results applied using the DenseNet- 121 pre-trained CNN, and GWO per class.

CNN	Class	Performance of the Classifier				
		Accuracy (%)	Sensitivity	Specificity	Precision	AUC
Before pre-processing (Softmax)	Benign	69.0	65.2	65.3	53.3	0.45
	Malignant	66.6	63.1	68.1	57.1	0.44
	Normal	62.6	60.7	72.2	60.2	0.45
	**Average**	**66.06**	**63.0**	**68.53**	**56.86**	**0.446**
After pre-processing (Softmax)	Benign	98.2	98.1	98.9	97.0	0.99
	Malignant	97.8	97.8	98.4	97.2	0.99
	Normal	97.9	98.5	98.2	98.1	0.99
	**Average**	**97.96**	**98.13**	**98.5**	**97.43**	**0.99**
After pre-processing (SVM)	Benign	99.3	99.1	98.9	96.6	1
	Malignant	99.7	99.6	98.7	97.1	0.99
	Normal	98.8	99.4	97.9	96.4	0.99
	**Average**	**99.26**	**99.36**	**98.5**	**96.7**	**0.99**
After pre-processing (RF)	Benign	98.7	98.9	99.1	97.6	0.99
	Malignant	98.1	99.1	98.3	98.3	0.99
	Normal	99.2	97.9	98.9	98.1	0.99
	Average	**98.66**	**98.63**	**98.76**	**98**	**0.99**

**Table 3:** The MIAS dataset results applied using the VGG-16 pre-trained CNN, and GWO.

CNN	Class	Performance of the Classifier				
		Accuracy (%)	Sensitivity	Specificity	Precision	AUC
Before pre-processing (Softmax)	Benign	69.19	62.1	69.2	62.1	0.45
	Malignant	67.82	68.4	69.9	64.2	0.45
	Normal	70.63	73.3	72.3	66.9	0.46
	**Average**	**69.21**	**67.93**	**70.46**	**64.4**	**0.45**
After pre-processing (Softmax)	Benign	98.82	97.2	99.1	97.1	1.0
	Malignant	98.1	98.1	98.5	97.8	0.99
	Normal	97.3	98.9	98.7	98.4	0.99
	**Average**	**98.06**	**98.06**	**98.76**	**97.76**	**0.993**
After pre-processing (SVM)	Benign	99.7	100	98.8	96.6	1.0
	Malignant	99.9	99.9	99.6	97.5	1.0
	Normal	100	99.8	99.7	97.2	1.0
	**Average**	**99.86**	**99.9**	**99.7**	**97.1**	**1.0**
After pre-processing (RF)	Benign	99.8	99.9	99.4	97.2	1.0
	Malignant	99.7	98.6	99.8	98.3	1.0
	Normal	99.2	99.5	99.2	97.9	1.0
	**Average**	**99.56**	**99.66**	**99.56**	**97.8**	**1.0**

**Table 4:** The INbreast dataset results applied using the DenseNet- 121 pre-trained CNN, and GWO.

CNN	Class	Performance of the Classifier				
		Accuracy (%)	Sensitivity	Specificity	Precision	AUC
Before pre-processing (Softmax)	Benign	65.2	45.2	69.3	51.3	0.45
	Malignant	66.6	43.1	70.1	49.1	0.46
	Normal	67.6	49.7	68.2	50.2	0.45
	**Average**	**66.46**	**46.0**	**69.2**	**50.2**	**0.45**
After pre-processing (Softmax)	Benign	97.9	98.3	98.9	97.3	0.99
	Malignant	98.1	98.0	98.1	97.0	0.99
	Normal	98.5	97.9	98.4	98.3	0.99
	**Average**	**98.16**	**98.06**	**98.46**	**97.5**	**0.99**
After pre-processing (SVM)	Benign	99.3	98.7	99.2	98.6	0.99
	Malignant	99.1	98.5	99.1	96.1	0.99
	Normal	99.5	99.1	99.3	97.9	1.0
	**Average**	**99.3**	**98.76**	**99.2**	**97.5**	**0.99**
After pre-processing (RF)	Benign	99.1	98.7	99.1	97.2	0.99
	Malignant	98.8	98.1	98.3	95.8	0.99
	Normal	99.3	98.9	98.9	97.2	0.99
	Average	**99.1**	**98.56**	**98.76**	**96.7**	**0.99**

**Table 5:** The INbreast dataset results applied using the VGG-16 pre-trained CNN, and GWO.

CNN	Class	Performance of the Classifier				
		Accuracy (%)	Sensitivity	Specificity	Precision	AUC
Before pre-processing (Softmax)	Benign	65.9	45.2	69.37	51.8	0.49
	Malignant	66.5	43.4	69.9	49.3	0.48
	Normal	68.1	49.8	68.4	51.1	0.49
	**Average**	**66.83**	**46.1**	**69.22**	**50.7**	**0.486**
After pre-processing (Softmax)	Benign	97.96	98.4	99.1	97.8	0.99
	Malignant	98.5	97.9	98.3	96.9	0.99
	Normal	98.7	98.1	98.6	98.9	0.99
	**Average**	**98.4**	**98.13**	**98.66**	**97.86**	**0.99**
After pre-processing (SVM)	Benign	99.4	98.8	99.3	97.9	1.0
	Malignant	99.2	98.9	98.9	96.2	1.0
	Normal	99.6	99.4	99.4	98.2	1.0
	**Average**	**99.4**	**99.03**	**99.2**	**97.4**	**1.0**
After pre-processing (RF)	Benign	98.9	98.9	98.7	97.3	1.0
	Malignant	99.3	98.2	98.4	96.1	0.99
	Normal	99.6	99.1	99.2	98.1	1.0
	**Average**	**99.3**	**98.7**	**98.8**	**97.2**	**0.996**

Tables 2 and 3 showed that results of the optimized DenseNet- 121 and VGG-16 with the GWO hybrid with the BiLSTM. The obtained features are classified using softmax, SVM, and RF classifiers. The illustrated results showed that importance of preprocessing for increasing the model performance. Tables 4 and 5 showed the results of the applied model on the INbreast dataset. The best results achieved using the VGG-16 pretrained CNN with overall accuracy of 99.4%. The results proved the importance of the proposed model on the BT classification improvement. The VGG-16 achieved the best results over the two applied datasets. The experimental analyses show that as network depth increases. a model’s generalizability decreases.

From the mentioned tables, it can be observed that the best performance was achieved with the SVM classifier. in both networks with average values of 99.86%, 99.9%, 99.7%, 97.1%, and 1.0, respectively for accuracy, sensitivity, specificity, precision, and area under the ROC curve (AUC) on the MIAS dataset and 99.4%, 99.03%, 99.2%, 97.4%, and 1.0, respectively on the INbreast dataset. The results of the related experiments are illustrated in Tables 6 and 7, where performance is contrasted to other currently available models. The evaluation results prove that in terms of accuracy, sensitivity, specificity, and AUC, the proposed model outperforms other current models.

**Table 6:** Comparison between related works and the presented model based on the MIAS dataset.

Method	Accuracy (%)	Sensitivity (%)	Specificity (%)	Precision (%)	AUC
Abbas [17]	91.50%	92%	84.20	-	0.91
Sha et al. [18]	92%	96%	93%	-	-
Charan et al. [19]	65%	-	-	-	-
Abeer et al. [27]	96.8%				
Abeer et al. [28]	98.96%	97.83%	99.13%	97.35%	0.995
Raaj et al. [33]	99.17%	98%	98.66%	-	-
El Houby et al. [34]	95.3%	98%	92.6%	-	0.974
**The proposed**	**99.86**	**99.9**	**99.7**	**98.16**	**1.0**

**Table 7:** Comparison between related works and the presented model based on the INbreast dataset.

Method	Accuracy (%)	Sensitivity (%)	Specificity (%)	Precision (%)	AUC
Abeer et al. [29]	97.1	96.3	97.9	-	0.988
Abeer et al. [30]	99.236	98.8	99.1	96	0.998
Akselrod-Ballin et al. [31]	78	-	-	-	-
Al-Antari et al. [32]	95.3	-	-	-	-
Singh et al. [35]	90.4	92.0	88.0	--	-
Chakravarthy et al. [36]	96.6	-	-	-	-
**The proposed**	**99.4**	**99.03**	**99.2**	**97.4**	**1.0**

## 5. Conclusion

This paper introduced an enhanced CNN model for BT detection and classification based on a hybrid structure of adapted CNN with BiLSTM. The MIAS mammographic dataset is applied for model evaluation. First, the mammographic data is pre-processed in five steps aiming to increase the model performance and reduce the training time. Then the enhanced CNN model is applied to enhance the BC mammography data classification. The pre-processed mammographic data features are extracted using the modified CNN such as DenseNet-121 and VGG-16 while the temporal features are extracted using BiLSTM. Finally, the softmax, SVM, and RF classifiers are applied to perform the classification task. The experimental analysis shows that the enhanced VGG-16 performs better than enhanced DenseNet-121 for BC classification using the SVM classifier with the overall accuracy, sensitivity, specificity, precision, and AUC values of 99.86%, 99.9%, 99.7%, 97.1%, and 1.0, respectively on the MIAS dataset and 99.4%, 99.03%, 99.2%, 97.4%, and 1.0, respectively on the INbreast dataset.
